# Concurrent Banff 2A Acute Cellular Rejection and BK Virus Nephropathy in a Kidney Transplant Recipient: A Case Report and Review of Management Strategies

**DOI:** 10.7759/cureus.85926

**Published:** 2025-06-13

**Authors:** Mojgan Jalalzadeh, Mingyu Cheng

**Affiliations:** 1 Internal Medicine/Transplant Nephrology, Baylor Scott & White Health, Temple, USA; 2 Pathology, Baylor Scott & White Health, Temple, USA

**Keywords:** acute cellular rejection, bk polyomavirus, delayed graft function, immunosuppression management, kidney transplantation

## Abstract

We present a complex case of a 71-year-old man with end-stage renal disease secondary to autosomal dominant polycystic kidney disease who developed acute Banff 2A cellular rejection in association with BK virus nephropathy following a deceased donor kidney transplant. Despite the initial delayed graft function and subsequent wound complications, the patient stabilized with appropriate immunosuppression and antiviral prophylaxis. This case highlights the challenges of balancing immunosuppression for the management of graft rejection while minimizing viral activation, with an emphasis on evidence-based management of BK polyomavirus.

## Introduction

BK polyomavirus (BKPyV) is a highly prevalent double-stranded DNA virus that infects 80-90% of the general population. Initial exposure usually occurs in childhood and is often asymptomatic [1]. The virus remains latent in renal tubular epithelial cells and urinary tract epithelium. However, in immunocompromised individuals, particularly transplant recipients, reactivation can occur, leading to BKPyV-associated nephropathy (BKPyVAN) and possible graft loss [2]. The highest incidence of BKPyVAN occurs in the first two to six months after transplantation. While most cases occur in the first year after transplantation, BKPyVAN can occur years after transplantation. Retrospective and prospective studies have described the typical course of BK virus disease after kidney transplantation: 30-60% of transplant recipients develop BK Viruria, 10-20% progress to viremia, and 5-10% ultimately develop BKPyVAN [3].

Acute graft rejection can occur in 8% to 12% of kidney transplant recipients with established Bk or BKPyVAN virus following a reduction of immunosuppression [4]. Renal allograft biopsy can be useful in diagnosing transplant rejection in these circumstances. These episodes are typically responsive to corticosteroid therapy.

T-cell-mediated graft rejection (TCMR), also known as acute cellular rejection, remains a common and important cause of allograft dysfunction, especially in the early post-transplant period. The disease is characterized by an immune response, in which recipient T lymphocytes recognize donor alloantigens, leading to infiltration of activated T cells into the graft and subsequent tubulitis, interstitial inflammation, and, in more severe cases, arthritis. The Banff classification provides a standard histopathological grading of TCMR, ranging from borderline to grades I, II, and III, with increasing severity of inflammation and tissue damage.

This case highlights the complex management of post-transplant complications in a patient with BKPyVAN and acute Banff 2A cellular rejection, requiring a careful balance between controlling graft rejection and managing opportunistic viral infections

## Case presentation

A 71-year-old male with end-stage renal disease due to polycystic kidney disease (autosomal-dominant polycystic kidney disease) who had been on peritoneal dialysis for four years underwent deceased donor renal transplant in September 2023.

Donor characteristics

The donor was a 55-year-old white male with a history of hypertension and intravenous drug abuse who died of head trauma. The final creatinine level was 3.98 mg/dL, and the kidney donor profile index (KDPI) was 87%. Pretransplant renal biopsy showed no significant glomerulosclerosis or pathology. Cold ischemia time was 15 hours and 3 minutes. Both donor and recipient were blood type O and seropositive for cytomegalovirus and Epstein-Barr virus. Basiliximab was used for induction therapy, as the recipient's calculated panel reactive antibody was 0%.

Postoperative period

The initial posttransplant period was complicated by delayed graft function (DGF) and required hemodialysis for two weeks. In addition, he developed a wound dehiscence at the graft site that required surgical repair. He was subsequently discharged in stable condition. Routine monitoring in December 2023 revealed BK viremia at 76,333 IU/mL. Immunosuppressive therapy was rapidly adjusted, initially by reducing the dose of mycophenolic acid, which was later discontinued. By late January 2024, rising serum creatinine and newly detected donor-specific antibodies prompted a graft biopsy (Table 1).

**Table 1:** Timeline BKPyVAN, BK polyomavirus-associated nephropathy; DSA, donor-specific antibody

Date	Event
Sep 2023	Deceased donor kidney transplant performed
Sep–Oct 2023	Delayed graft function; hemodialysis for 2 weeks
Oct 2023	Wound dehiscence: surgical repair performed
Dec 2023	BK viremia (76,333 IU/mL) detected
Jan 2024 (early)	Mycophenolic acid dose reduced, then discontinued
Jan 2024 (late)	Rising serum creatinine from 1 mg/dL to 1.68 mg /dL, and new DSAs detected
Feb 2024	Graft biopsy: Banff 2A acute cellular rejection + findings suggestive of BKPyVAN
Feb 2024	IV methylprednisolone started; oral prednisone tapered
Apr 2025	Serum creatinine 1.40 mg/dL; BK viremia decreased to 8,800 IU/mL

**Figure 1:**
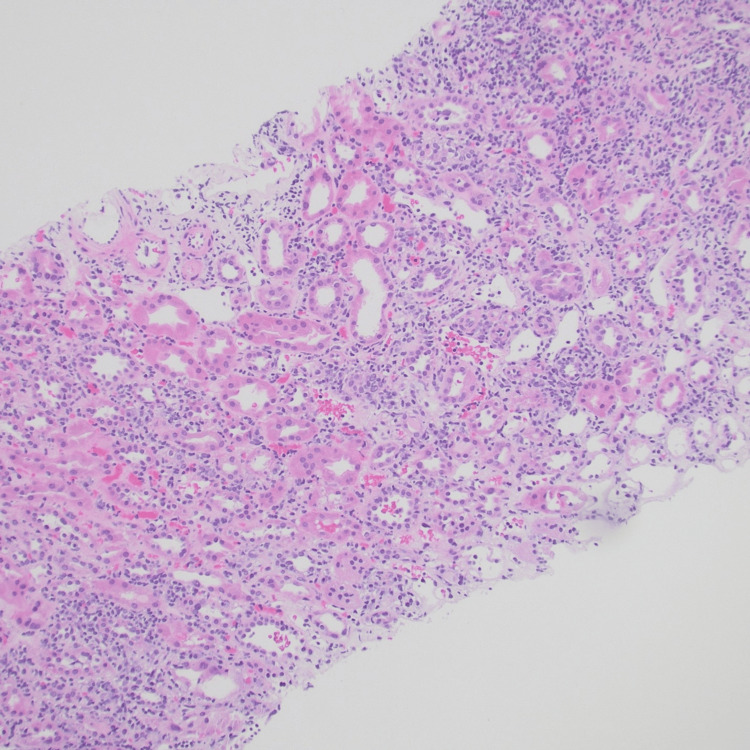
Biopsy showing widespread interstitial inflammation and acute tubular injury in the cortex. Tubulitis is also present (H&E).

**Figure 2:**
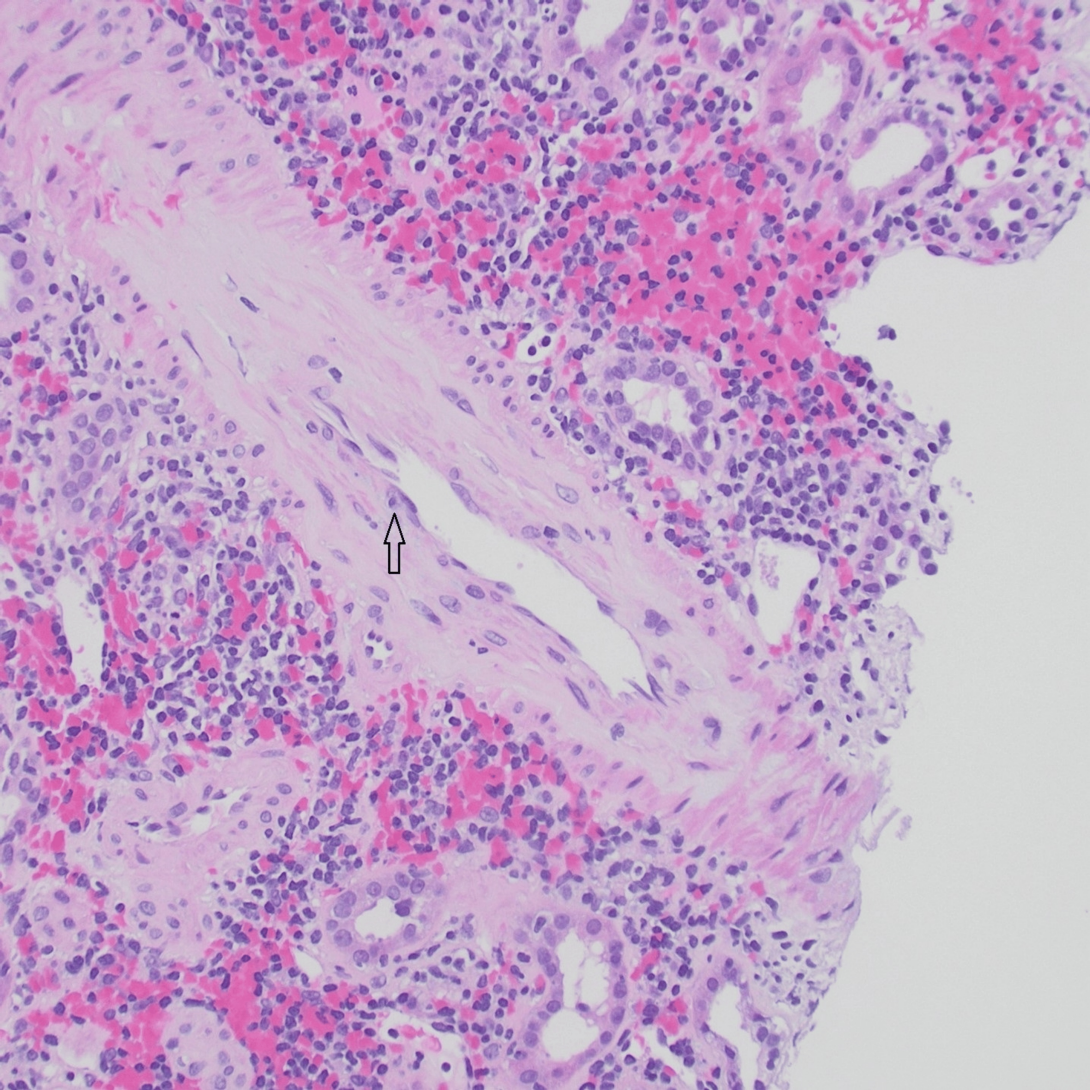
The artery showing mild endotheliitis (H&E).

**Figure 3:**
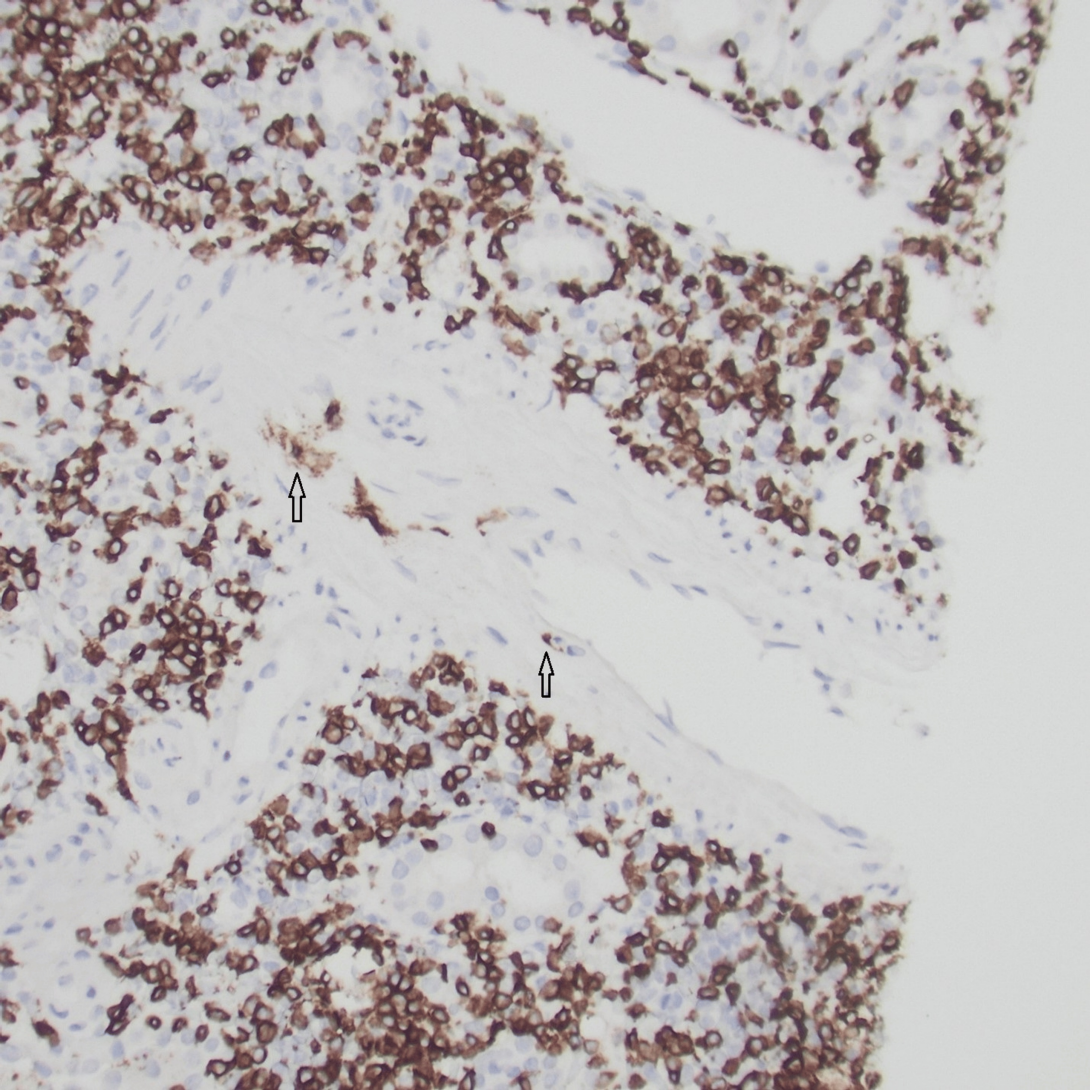
Immunohistochemical stain for CD3 highlights interstitial infiltrate of T-cells and endotheliitis (arrow).

**Figure 4:**
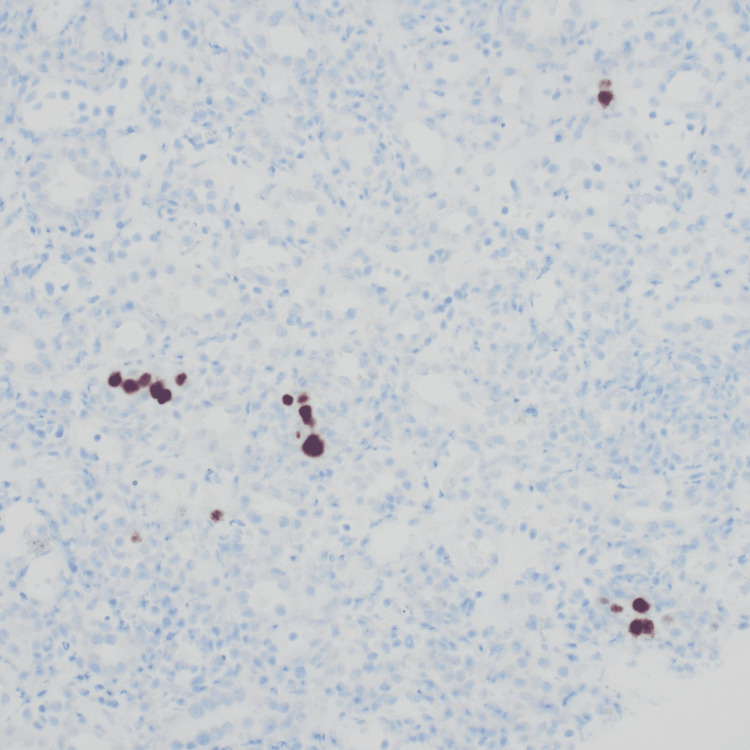
Immunohistochemical stain for SV40 showing positive staining in scattered tubular epithelial nuclei.

Histopathology revealed extensive medullary inflammation, acute Banff 2A cellular rejection, and features suggestive of BK virus nephropathy (Figures 1-4). Although no definitive viral inclusions were seen, significant interstitial inflammation and vascular involvement suggest active rejection rather than isolated BKPyVAN.

Management

The immunosuppressive regimen was carefully adjusted to manage the risk of allograft rejection and BK virus replication. The patient was treated with high-dose intravenous methylprednisolone (500 mg daily for three days), followed by oral prednisone at a dose of 60 mg daily. The prednisone dose was then gradually tapered over two weeks to a maintenance dose of 5 mg daily. Mycophenolic acid was discontinued, and the patient's tacrolimus levels were maintained between 4 and 6 ng/mL.

Follow-up

**Figure 5:**
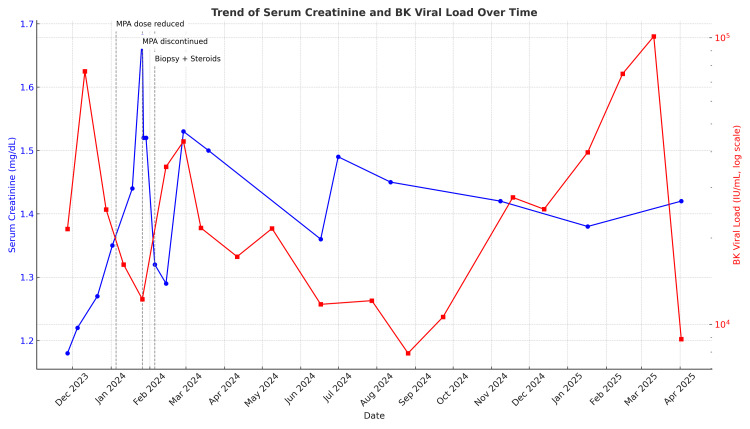
Trends in serum creatinine and BK viral load over time, along with therapeutic interventions. The blue line indicates serum creatinine (mg/dL), the red line indicates BK viral load (log scale, international units per milliliter), the vertical dashed lines and labels indicate ↓MPA dose, MPA discontinuation, biopsy + start intravenous steroids.

As of April 2025, the patient remained clinically stable, with a serum creatinine level of 1.40 mg/dL, an estimated glomerular filtration rate (GFR) of 53 mL/min, and a BK virus PCR level in the blood of 8,800 IU/mL. Changes in serum creatinine and BK viral load over time, along with therapeutic interventions, are shown in Figure 5.

## Discussion

This case illustrates the complex interplay between immunosuppression and infection risk in kidney transplantation, particularly when acute rejection and BK virus infection co-occur. Immunosuppressive therapy is a known risk factor for BKPyV reactivation, particularly in the first year after transplantation. Calcineurin inhibitors (CNIs), particularly tacrolimus, and antimetabolites, such as mycophenolate mofetil, increase susceptibility [5]. Other risk factors include donor-recipient mismatch (particularly BK-positive donors and BK-negative recipients), DGF, placement of a ureteral stent, and prior severe immunosuppression for episodes of rejection [5]. In this patient, risk factors included advanced age, DGF, and aggressive immunosuppressive therapy. Interestingly, some protective factors, such as the use of mTOR inhibitors (e.g., everolimus) [6], HLA-B51 positivity [7], and ADPKD [8], have been associated with lower risks of BKPyV reactivation.

Pathogenesis

BKPyV reactivation is caused by T-cell-mediated immunodeficiency, which reduces viral control and enhances replication [9]. The resulting damage to renal tubular epithelial cells leads to inflammation, tubular atrophy, interstitial fibrosis, and progressive nephron loss. Continued viral replication can stimulate immune responses that compromise graft function and contribute to acute graft rejection [10].

Clinical manifestations

BKPyV infection may present as asymptomatic viruria and sometimes progress to viremia or BKPyVAN [11] and gradually impair graft function [11]. Less commonly, it may cause hemorrhagic cystitis and has been associated with genitourinary tract malignancies [12].

Diagnostic approach and screening

Routine monitoring by quantitative plasma PCR is crucial for early diagnosis and management. The threshold for plasma viral load for BKPyV to be considered positive or clinically significant varies depending on the specific assay used. In general, levels >1,000 copies/mL are considered positive in most assays, and levels >10,000 copies/mL correlate to biopsy-confirmed BKPyVAN. Guidelines recommend monthly testing for the first nine months after transplantation, quarterly testing from months 9 to 24, and annual testing thereafter, or based on graft function indices [3]. Renal biopsy remains the gold standard for the diagnosis of BKPyVAN, demonstrating the presence of viral inclusions, tubulointerstitial inflammation, and positivity for SV40 antigen [13]. Urine cytology and detection of Decoy cells serve as adjunctive, albeit less specific, diagnostic tools [14].

Management strategies

Reduction of immunosuppression remains the cornerstone of BKPyV therapy [3]. Antimetabolites should be reduced by 50% and discontinued if necessary [3]. In refractory cases, CNIs should be reduced by 25-50% [15]. Adjuvant therapies include intravenous immunoglobulin, especially in refractory cases or patients with hypogammaglobulinemia [16]. Novel therapies, such as BKPyV-specific T-cell therapy, are under active investigation [17].

Prevention

Preventive strategies emphasize regular monitoring and individualized immunosuppressive regimens. Avoiding over-inducible immunosuppression in low-risk patients, optimizing maintenance regimens, and closely monitoring high-risk patients in the first year after transplantation are essential [18].

Prognosis

With prompt and appropriate intervention, viremia can often be resolved and graft function preserved. However, delay in treatment can lead to BKPyVAN and graft loss in 15-50% of cases [19].

Special considerations

The management of BKPyVAN and acute graft rejection is particularly challenging. Initial anti-rejection therapy, usually corticosteroids, should be followed by careful and gradual tapering of immunosuppression [20]. In this patient, careful adjustment of immunosuppression resulted in a reduction in BK virus burden and stable renal function (GFR 59 mL/min). Re-transplantation usually has favorable outcomes when the virus has been cleared before surgery.

## Conclusions

This case highlights the importance of careful monitoring and personalized immunosuppression strategies in kidney transplant recipients. The coexistence of acute cellular rejection and BK virus infection requires a careful and balanced approach to effectively manage graft rejection while controlling viral replication to achieve optimal transplant outcomes. Continued research into the pathogenesis and management of BKPyV is critical to improving long-term graft survival.
